# Locations that Support Social Activity Participation of the Aging Population

**DOI:** 10.3390/ijerph120910432

**Published:** 2015-08-26

**Authors:** Pauline van den Berg, Astrid Kemperman, Boy de Kleijn, Aloys Borgers

**Affiliations:** Eindhoven University of Technology, P.O. Box 513, 5600MB Eindhoven, The Netherlands; E-Mails: a.d.a.m.kemperman@tue.nl (A.K.); boydekleijn@hotmail.com (B.K.); a.w.j.borgers@tue.nl (A.B.)

**Keywords:** social contact, interaction, elderly, living environment, amenities, latent class

## Abstract

Social activities are an important aspect of health and quality of life of the aging population. They are key elements in the prevention of loneliness. In order to create living environments that stimulate older adults to engage in social activities, more insight is needed in the social activity patterns of the aging population. This study therefore analyzes the heterogeneity in older adults’ preferences for different social activity location types and the relationship between these preferences and personal and mobility characteristics. This is done using a latent class multinomial logit model based on two-day diary data collected in 2014 in Noord-Limburg in the Netherlands among 213 respondents aged 65 or over. The results show that three latent classes can be identified among the respondents who recorded social activities in the diary: a group that mainly socializes at home, a group that mainly socializes at a community center and a group that is more likely to socialize at public ‘third’ places. The respondents who did not record any interactions during the two days, are considered as a separate segment. Relationships between segment membership and personal and mobility characteristics were tested using cross-tabulations with chi-square tests and analyses of variance. The results suggest that both personal and mobility characteristics play an important role in social activity patterns of older adults.

## 1. Introduction

The ageing of the population poses challenges for urban and transport planners to create living environments that support older adults’ health and quality of life. This implies that opportunities for social contacts should be created that are accessible for older adults. Compared to their younger counterparts, older people are in general less mobile, spend more time at home, and participate in fewer social settings (e.g., work, school or sports clubs), which probably makes them more reliant on their neighborhood for social contacts. Neighborhood services and facilities such as shops, sports facilities, cafés and restaurants, community centers, and recreational areas can provide opportunities for social contacts among residents. However, the number of local facilities is decreasing due to increasing mobility, regionalization, Internet-use, and demographic decline in certain areas (e.g., Oldenburg, 1989 [1]; Hickman, 2013 [2]; CBS, 2015 [3]). This may have negative outcomes for the social participation of older adults and consequently their quality of life. A lack of social participation may result in social isolation and loneliness, which may cause a reduction in physically activity, and both mental and physical health (British Columbia, 2014 [4]). In order to promote healthy aging it is crucial to stimulate social contacts among older adults. Creating environments that support social participation of the ageing population is therefore an important challenge for urban and transport planners.

For successful urban and transport planning for the aging population, more insight is needed in seniors’ choices for social activity locations and the extent to which social contacts take place at different (local) facilities. The aim of this study therefore is to analyze the diversity of older adults’ preferences for different social activity location types and the relationship between these preferences and personal and mobility characteristics. This is done by estimating a latent class multinomial logit model of individuals’ social interaction location choices based on data collected among 213 respondents aged 65 or over collected in 2014 in several towns in Noord-Limburg in the southeast of the Netherlands.

This article first describes the existing literature on social activity patterns of older adults. Next, the data collection and analysis methods are described, followed by the description of the results. The article finishes with a discussion of the findings and the implications for urban and transport policy.

## 2. Literature

Many countries all over the world are experiencing a sharp rise in the ageing population. Ageing is in general associated with decreased health, mobility and social participation, and changes in the size and composition of one’s social network (e.g., through retirement or loss of a partner), which impacts activities such as traveling and socializing, and consequently, quality of life. Several studies in transportation research have focused on travel behavior of older adults (e.g., Tacken, 1998 [5]; Alsnih and Hensher, 2003 [6]; Silvis and Niemeier, 2009 [7]; Mercado and Páez, 2009 [8]; Mohammadian *et al.*, 2013 [9]; Horner *et al.*, 2015 [10]). The literature shows that the elderly have become more mobile compared to earlier generations in terms of travel frequency, distance (e.g., Rosenbloom, 2001 [11]; Collia *et al.*, 2003 [12]; Páez *et al.*, 2006 [13]) and car ownership (Schmöcker *et al.*, 2005 [14]; Bannister and Bowling, 2004 [15]; Newbold *et al.*, 2005 [16]). Among the elderly, the car is the preferred mode (Kim and Ulfarsson, 2004 [17]; Collia *et al.*, 2003 [12]; Kemperman and Timmermans, 2009 [18]), followed by walking. In the Netherlands, a relatively large share of elderly trips is made by bicycle (Tacken, 1998 [5]; Kemperman and Timmermans, 2009 [18]; Van den Berg *et al.*, 2011 [19]). However, the travel frequency and distance of older people are still significantly lower compared to those of younger people. This holds especially for the oldest group of elderly, for women and elderly with walking disabilities (e.g., Collia *et al.*, 2003 [12]).

Older people also perform fewer social activities (Aoki *et al.*, 1996 [20]; Páez *et al.*, 2006 [13]; Kemperman *et al.*, 2006 [21]; Carrasco and Miller, 2006 [22]; Farber and Paéz, 2009 [23]) and have a smaller social network than their younger counterparts (e.g., Van den Berg *et al.*, 2009 [24]). As a result, seniors have a heightened risk of social isolation and loneliness (e.g., Hawkley *et al.*, 2008 [25]; Scharf and De Jong Gierveld, 2008 [26]; De Jong Gierveld and Van Tilburg, 2010 [27]; Pino *et al.*, 2014 [28]; Weijs-Perrée *et al.*, 2015 [29]; Van den Berg *et al.*, in press [30]), which in turn carries the risk of being less physically active, and having reduced mental and physical health (British Columbia, 2014 [4]). In order to reduce these risks it is crucial to stimulate older adults’ social participation.

Social networks can provide people with access to instrumental resources and emotional support. Social network ties can be developed and maintained through face-to-face social interaction or communication through different ICTs. Studies investigating the relationship between face-to-face and ICT-mediated social interactions have shown that ICT-mediated contacts complement face-to-face contacts (e.g., Mokhtarian *et al.*, 2006 [31]; Frei and Axhausen, 2008 [32]; van den Berg *et al.*, 2012 [33]; Carrasco, 2011 [34]). However, the elderly still lag behind in the use of communication technology. Moreover, according to the study by Cummings *et al.* (2002) [35] face-to-face social interactions are more useful than digital interactions for developing and sustaining social relationships. This study therefore focuses on face-to-face social contacts.

Older adults particularly rely on their neighbourhood for social contacts because they spend more time at home, are less mobile and participate in fewer other settings such as work, school or sports clubs (e.g., Völker and Flap, 2007 [36]; van den Berg *et al.*, forthcoming [37]). Policymakers are currently stimulating ageing in place, which is defined as “the ability to live in one’s own home and community safely, independently, and comfortably, regardless of age, income, or ability level.” (Centers for Disease Control and Prevention, 2014 [38]). For ageing in place to be successful, neighborhoods should meet the needs of older adults and provide access to face-to-face social activities.

The aging of the population poses challenges for urban and transport planners to provide sufficient levels of accessibility for older adults. The concept of accessibility deals with the activity locations in a given area and the effectiveness with which people can reach these, given the available transportation system (Horner *et al.*, 2015 [10]). Accessible local facilities may provide opportunities for social interaction and expansion and maintenance of local social networks (e.g., Talen, 1999 [39]; Oldenburg, 1989 [1]; Dempsey *et al.*, 2012 [40]; Francis *et al.*, 2012 [41]; Hickman, 2013 [2]). Horner *et al.* (2015) [10] studied the differences in accessibility to activities and facilities among different age groups. Their findings indicate that the youngest age group (18–49) has the highest level of accessibility. Older adults have lower accessibility, with the lowest level for the middle age group (50–64). The finding that the oldest age group does not have the lowest access to facilities is probably related to the fact that retirement homes tend to cluster around (health care) facilities.

In a previous study we found accessibility, mobility and satisfaction with the facilities in the neighborhood can decrease feelings of loneliness (van den Berg *et al.*, 2015 [30]). However, the number and quality of local services and facilities such as shops, schools, sports facilities, cafés, community centers, libraries and recreational areas has decreased over the last decades (e.g., Oldenburg, 1989 [1]; Hickman, 2013 [2]; CBS, 2015 [3]). For instance, in the Netherlands the number of facilities per 1000 inhabitants has decreased with 8% between 2001 and 2005. The number of food shops per 1000 inhabitants even decreased with 18% and the number of banking offices decreased with 38% (CBS, 2015 [3]). There are a number of reasons for this trend. Increasing mobility and increasing use of the Internet (e-commerce) have lowered the demand for local facilities. In addition, economic recession seems to aggravate this trend (Hickman, 2013 [2]). This trend may hamper older adults’ social participation, as local public facilities have been found to be important for social interaction.

Hickman (2013) [2] studied the role of public facilities for social interaction in deprived neighborhoods in Great Britain based on 180 in-depth interviews. He found that residents particularly value shops for social interaction. Parks and community centers were also valued, especially by people who spent more time at home, such as the elderly. Francis *et al.* (2012) [41] studied the role of public facilities for creating sense of community in Perth, Australia. Analyzing survey data from 911 respondents they found that shops and public open space contribute to sense of community. They found community centers and schools to be less relevant. Whalen *et al.* (2012) [42] also found that shops and services in the neighborhood are important for creating a sense of community. Based on social interaction diary data collected from 747 respondents in the Eindhoven area, Van den Berg *et al.* (2014) [43] estimated a latent class model on social interaction location choice. Their results indicated that a relatively large segment mainly performed social activities at home. A second segment preferred social interactions at work. A third segment was more likely to have social contacts at public space outdoors and school or childcare facilities, while the fourth segment preferred cafés, restaurants, and sports facilities. Segment membership was explained by personal and household characteristics such as gender, age, household type, number of face-to-face social interactions, and frequency of contact with neighbors, as well as characteristics of the residential environment such as urban density, distance to several facilities (supermarket, family doctor and community center) and satisfaction with local facilities.

Although the discussed studies acknowledged the importance of public facilities for social interaction, they did not focus specifically on the importance of these facilities for the elderly. For successful urban design and planning for the growing aging population, more knowledge is needed on their spatial choices for social activities. When studying senior citizens and their social activity patterns, their heterogeneity should be incorporated. Their difference in social activity patterns may be reflected by differences in personal and household characteristics, characteristics of the residential location and the use of different transport modes. Therefore, this article presents the results of a latent class multinomial logit model of older adults’ social interaction location choices, followed by the results of bivariate analyses used to analyze the links between latent class membership and several personal, residential and mobility attributes.

## 3. Methods

### 3.1. Data Collection

The data for this study were collected using a two-day social interaction diary and a questionnaire. In the diary respondents were asked for a number of different types of locations, whether they had visited that type of location, and whether they had had contact with other persons at that location. For each contact they were asked to indicate start and end time, whether the interaction was preplanned, routine or spontaneous, with whom they interacted, the purpose of the interaction, and how important the interaction was for them. The questionnaire was used to collect data on personal and mobility characteristics.

**Figure 1:**
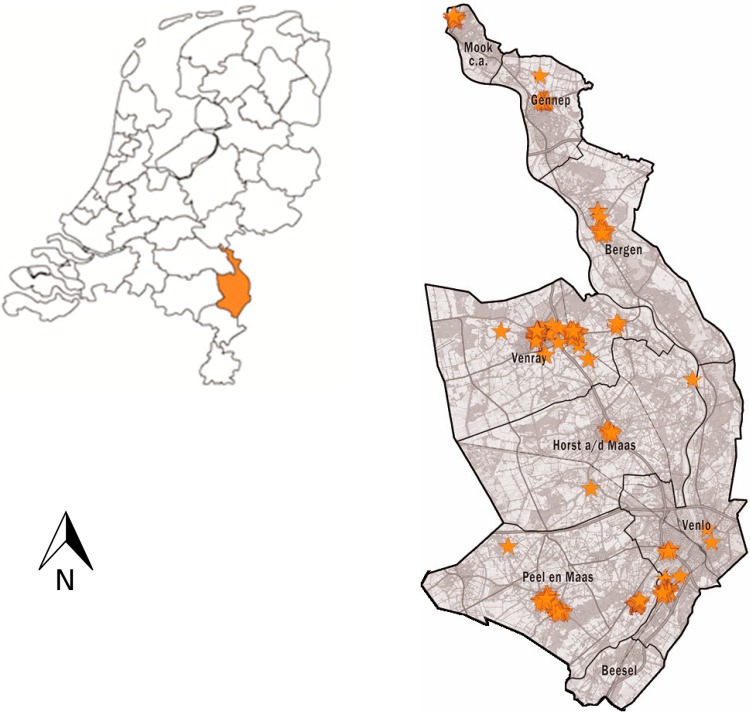
Map of Noord-Limburg and sampling locations.

The data were collected in several towns in Noord-Limburg, which is located in the southeast of the Netherlands. Noord-Limburg, the northern part of the province Limburg, consists of the municipalities Beesel, Bergen, Gennep, Horst aan de Maas, Mook en Middelaar, Peel en Maas, Venlo and Venray (see Figure 1). The area inhabits 280,600 people, out of whom 18% is 65 or over. The area is expecting a demographic decline and increased ageing, which is expected to result in 3% less inhabitants in 2030, out of whom 28% will be 65 and over. This percentage is higher than the expected percentage of older adults in the Dutch population (24%) in 2030. This makes Noord-Limburg an interesting study area for this research.

In order to have variation with respect to the living environment, data were collected in areas that differed with respect to urban density. The degree of urbanization was measured based on the “surrounding address density”, which is the average number of addresses per 500 meter square within a kilometer radius from the address. This indicator has been widely used in the Netherlands and consists of five categories: very strongly urbanized (surrounding address density of 2500 and over per km^2^), strongly urbanized (surrounding address density between 1500 and 2500 per km^2^), moderately urbanized (surrounding address density between 1000 and 1500 per km^2^), little urbanized (surrounding address density between 500 and 1000 per km^2^), and not urbanized (surrounding address density below 500 per km^2^). In Noord-Limburg, only 2% of the inhabitants live in a very high urban density area, namely in the center of Venlo. This highest density class is therefore merged with the second class. Equal numbers of diaries were distributed in each of the four density classes.

The data were collected between January and March 2014 using a deliver and pick up procedure, which resulted in a good response. Eighty-eight percent of the accepted diaries were returned and useful for the analyses. A total of 213 social interaction diaries were collected among respondents 65 or over.

### 3.2. Latent Class Multinomial Logit Model

To analyze the heterogeneity in social interaction location choices among older adults a latent class multinomial logit model is used (Greene 2002) [44]. This model groups the respondents into segments (latent classes). For each segment separate parameters are estimated showing their preferences for social interaction locations. The respondents who did not record any interactions are not included in the model. They are considered as a separate segment. The model accounts for the hierarchical structure of the data in which the number of social activities may very across respondents.

A fixed number of S segments is assumed to exist in the population. Given that an individual belongs to latent class s (s = 1,…., S), the utility function for individual i for social interaction location j on choice occasion t can be expressed as follows,

(1) $$U_{ijt}^{s}=\beta_{s}^{\prime }X_{ijt}+\varepsilon_{ijt}$$

where Xijt expresses all social interaction location type alternatives and β_s^' is a parameter vector for each latent class s. ɛijt is an error term representing unobserved heterogeneity for individual i and location type j (j = 1,….,J) at occasion t. For each segment or latent class the choice probabilities are generated, resulting in the latent class multinomial model:

(2) $$P\left(j\text{|}segment=s\right)=\frac{\text{exp}\left(\beta_{s}^{\prime }X_{ijt}\right)}{\sum_{j\prime =1}\text{exp}\left(\beta_{s}^{\prime }X_{ij\prime t}\right)}$$

The latent class specific probabilities are a set of fixed constants that sum to one. The multinomial logit (MNL) model estimates the probability of individual i belonging to latent class s:

(3) $$P\left(y_{ij}=j\right)=\sum_{s=1}^{S}P\left(segment=s\right)\left[\frac{\exp\left(\beta_{s}^{\prime }X_{ijt}\right)}{\sum_{j\prime =1}\exp\left(\beta_{s}^{\prime }X_{ij\prime t}\right)}\right]$$

Each individual is then assigned to the latent class with the highest probability. The latent class parameters can be estimated using maximum likelihood estimation. The goodness of fit of the model is indicated by McFadden's rho square (*ρ2 = 1–LL(β)/LL(0)*). The likelihood ratio test is used to check whether the estimated model performs better than the base model. This is the case if the *X^2^* value is higher than the critical *X^2^* value.

The relationship between latent class membership of the respondents and their personal and household characteristics, and mobility characteristics is tested using cross-tabulations (with Chi-square tests) and analysis of variance (with F ratio tests).

## 4. Results

### 4.1. Sample Characteristics

The sample consists of 213 respondents aged 65 or over. The characteristics of the sample are summarized in Table 1. As can be seen, the sample contains more women than men. One third of the sample is between 65 and 69 and a quarter of the respondents are 80 or over. Regarding household composition, the results show that more than one third of the respondents are single and the other two thirds live with a partner. Almost half of the respondents are lower educated. Ten percent of the respondents is severely physically challenged, and another 21% is somewhat physically challenged. As can be seen, the sample is roughly evenly distributed over the four classes of urban density.

### 4.2. Social Activity Characteristics

Out of the 213 respondents, 22 (10%) did not record any face-to-face social interactions in two days. The other 191 recorded a total of 1029 social interactions. The maximum number of interactions recorded in two days was 15. Overall, the average number of interactions in two days is 4.83, with a standard deviation of 3.28. The distribution of number of interactions can be seen in Figure 1.

**Table 1:** Sample characteristics (N = 213).

Variable	Levels	N	%
Gender	Male	91	43
	Female	120	57
Age	65–69	74	35
	70–74	44	21
	75–79	43	20
	80+	52	24
Household composition	Single	79	37
	Couple	134	63
Education	Primary	95	45
	Medium	51	24
	High (BSc or higher)	59	28
Gross yearly household income	<€21,000	34	16
	€21,000–€34,000	71	33
	€34,000–€43,000	51	24
	>€43,000	34	16
Physically challenged	Yes, severely	21	10
	Yes, somewhat	44	21
	No	143	67
Urban density	High (>1500 addresses/km^2^)	62	29
	Moderate (1000–1500 addresses/km^2^)	66	31
	Low (500–1000 addresses/km^2^)	45	21
	Very low (<500 addresses/km^2^)	40	19

**Figure 2:**
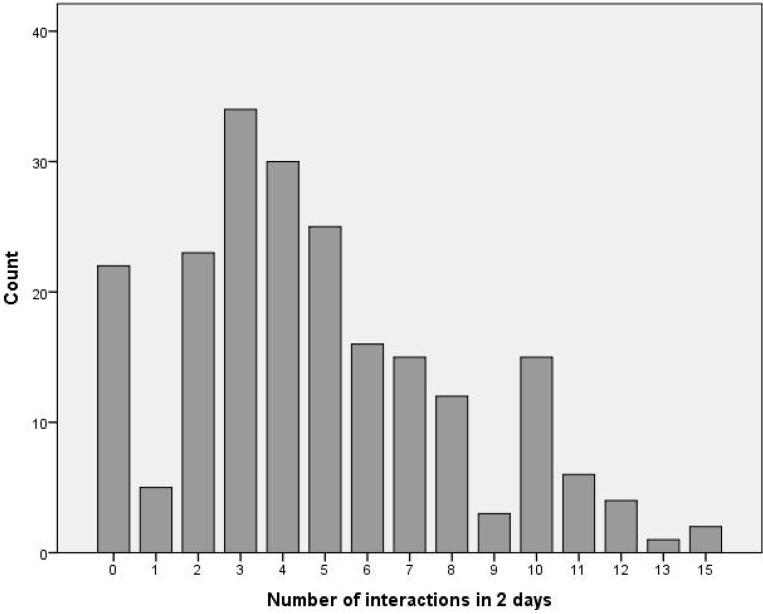
Number of recorded interactions in two days (N = 213).

Table 2 shows the distribution of the 1029 social interactions across types of location. On the left it shows the 19 different location types as recorded in the diary. For the analyses the location types were recorded into seven categories, which are shown on the right. As can be seen, home is the most common location for social interaction, followed by home of another person. In total, over one third of social interactions took place at a home location. Almost 15% of the interactions took place in public space outdoors, including parks and play facilities. The categories community center and church were merged. Together they account for 11% of the social interactions. This percentage is high compared to Van den Berg *et al.* (2014) [43], indicating that community centers are specifically important for older adults in less urbanized areas. Different types of shopping facilities were merged with health facilities. The category shop/services contains 18% of the social interactions. Five percent of the interactions took place at a bar or restaurant and 6% took place at a sports facility. As older adults typically do not work or study anymore, social interactions at work or school make up only a small part of social interactions. For this reason, these locations were merged with the category “other”.

The respondents were asked for each social interaction to indicate on a five point scale how important the interaction was to them. Table 3 shows the mean scores of importance of interactions for each type of location. As shown, social interactions at a bar or restaurant have the highest score on importance, followed by interactions at home. Social interactions at a community center or church have the lowest importance.

**Table 2:** Distribution of social interactions across types of location.

Recorded in Diary Type of Location	N	%		Used in LCModel Type of Location	N	%
Home	194	18.9		Home location	367	35.7
Home other person	173	16.8				
On the road	25	2.4		Public space/park	153	14.9
Public space outdoors	84	8.2				
Park	23	2.2				
Play facility	21	2.0				
Community center	94	9.1		Community center/church	117	11.4
Church	23	2.2				
Supermarket	84	8.2		Shop/services	186	18.1
Local shop	21	2.0				
Shopping center	44	4.3				
Health facility	37	3.6				
Bar	17	1.7		Bar/restaurant	46	4.5
Restaurant	29	2.8				
Sports facility	62	6.0		Sports facility	62	6.0
Library	2	0.2		Other	98	9.5
Work	11	1.1				
School	22	2.2				
Other	63	6.1				
Total	1029	100		Total	1029	100

**Table 3:** Mean importance of interactions per location type.

Type of Location	Importance (1–5)
Home location	4.01
public space /park	3.85
Community center/church	3.47
Shop/services	3.88
Bar/restaurant	4.38
Sports facility	3.72
Other	3.73
Total	3.82

F = 14.532, *p* < 0.000.

### 4.3. Latent Class Multinomial Logit Model

To analyze how the use of social interaction locations differs between respondents we use a latent class multinomial logit (MNL) model. Table 4 shows the estimation results of the latent class model as well as the conventional MNL model. The likelihood ratio test shows that the X^2^ value exceeds the critical X^2^ value which indicates that the estimated model performs better than the base model. Parameters are estimated for the categories: public space/park, shop/services, community center/church, bar/restaurant, sports facility and other type of location. The category “home location” serves as the reference category, which means that the estimated parameters should be interpreted relative this location type. The parameters for the conventional MNL model show the average likelihood for each location and are included for reasons of comparison. The parameters for the latent class MNL model indicate whether a specific segment is more or less likely than average to socialize at a certain type of location.

Three segments were identified, showing different parameters for social interaction locations. The first segment can be labeled as home socializers. This segment mainly has social contacts at a home location, which can be their own, or another person’s home. This segment contains 139 (65%) respondents. The second segment is described as third place socializers and contains 18 (9%) respondents. This segment is less likely to socialize at home and more likely than average to socialize at all other six types of locations. Shops and services have the highest parameter, followed by public space or park. The third segment, consisting of 34 (16%) respondents, can be labeled community center socializers, as they are more likely than average to socialize at a community center or church. The remaining 22 (10%) respondents who did not record any interactions make up a fourth segment which is labeled non-socializers. This segment is not included in Table 4. It will however be included in the next analyses.

**Table 4:** Modeling results.

Variable	Conventional MNL Model		Latent Class Model					
			Home Socializers		Third Place Socializers		Community Center Socializers	
	B	Sig.	B	Sig.	B	Sig.	B	Sig.
Public space/park	−0.875	0.000	−1.055	0.000	1.785	0.000	−1.102	0.000
Shop/service	−0.680	0.000	−1.068	0.000	2.494	0.000	−0.674	0.004
Community center	−1.143	0.000	−2.244	0.000	−0.638	0.364	0.825	0.000
Bar/restaurant	−2.077	0.000	−2.768	0.000	0.774	0.136	−0.815	0.001
Sports facility	−1.778	0.000	−1.885	0.000	0.889	0.068	−3.434	0.000
Other	−1.320	0.000	−1.256	0.000	−0.604	0.345	−2.734	0.000
Segment probability			0.73		0.09		0.18	
Log likelihood function *LL(*β*)*	−1790.082		−1714.367					
Restricted log likelihood *LL(0)*	−2002.342		−2002.342					
Likelihood ratio test	Χ^2^ = 151.43		Df = 12		Critical Χ^2^ (*p* = 0.05) = 21.03			
McFadden R^2^	0.106		0.144					

### 4.4. Effect of Personal, Household and Mobility Characteristics

Relationships between segment membership and personal and mobility characteristics were tested using cross-tabulations with chi-square tests and analyses of variance. The results are shown in Table 5. The results show no significant differences between segments with respect to gender. Regarding age we do find a significant difference between segments. The non-socializers are the oldest group, with an average age of almost 78, followed by the community center socializers with an average age of 76.5. The third place socializers are the youngest group with an average age of almost 70.

Regarding income and education level, significant differences between the segments are found as well. The third place socializers are more likely to have a higher income and a higher education. The community center socializers have the lowest income and 63% only has primary education. The non-socializers have the highest share (77%) of low educated respondents. This is also related to the fact that this segment has the highest average age. The segments are also found to significantly differ with respect to household composition. The third place socializers are most likely to live with a partner, whereas the non-socializers are most likely single.

Urban density was also found to have a significant relationship with latent class membership. The results show that the highest share of non-socializers live in high urban density areas. The community center socializers are most likely to live in moderate density areas and the third place socializers are most likely to live in low urban density areas. These findings are in line with the idea that people living in more dense areas are less social in the sense that they have a smaller or less strong social network and fewer social interactions (e.g., Aoki *et al.*, 1996 [20]; Van den Berg *et al.*, 2009 [24]). These findings may also be related to the fact that the oldest and least mobile group of elderly tend to live in old age homes which are located in higher density areas in town centers. This corresponds with the findings of Horner *et al.* (2015) [10] indicating that the oldest age group has higher access to facilities as retirement homes tend to cluster around health care facilities. While more facilities are present in higher density areas, the frequency of use is lower. Thus, the presence of facilities alone does not guarantee its use by the elderly.

The results show that the home socializers are most likely to volunteer. The non-socializers are least likely to volunteer, followed by the third place socializers. As expected, the average number of social interactions recorded in the two-day diary, differs significantly between the segments. The non-socializers did not record any social interactions. The third place socializers recorded the highest number of social interactions, with an average of six interactions in two days. In line with these results, the average score for loneliness is highest for the non-socializers and lowest for the third place socializers. The same pattern is found with respect to perceived health. The non-socializers have the lowest score on perceived health and are more likely to be physically challenged, whereas the third place socializers score highest on self-rated health and are less likely to be physically challenged.

With respect to mobility, respondents were asked which transport modes they use. The results again show significant differences between the segments. The non-socializers are less likely than average to walk, cycle, drive a car and use public transport. The third place socializers have the highest shares of users for all four transport modes. This indicates that mobility plays an important role in older adults’ social activity patterns.

Note that several variables in Table 5 are interrelated. For instance, higher age is associated with lower perceived health (r = −0.168; *p* < 0.05) and fewer social interactions (r = −0.354; *p* < 0.01). Self-perceived health is negatively correlated with feelings of loneliness (r = −0.267; *p* < 0.01), indicating that poor health increases the likelihood of feeling lonely. Health is also related to mobility. Perceived health is higher for people who walk, cycle, use a car and use public transport. The difference in health rating is largest for car use.

**Table 5:** Relationships between segments and personal, household and mobility characteristics.

Variable	1	2	3	4	Total	X^2^ or F (sig.)
	Home Socializers (N = 139)	Third Place Socializers (N = 18)	Community Center Socializers (N = 34)	Non-Socializers (N = 22)	(N = 213)	
**Gender**						
Male (%)	43	33	53	36	43	2.450 (0.484)
Female (%)	57	67	47	64	57	
**Age**						
Years mean	73.47	69.94	76.50	77.77	74.10	6.815 (0.000)
**Gross yearly household income**						
< €21,000 (%)	13	0	46	25	18	25.159 (0.000)
€21,000–€34,000 (%)	42	28	25	31	37	
> €34,000 (%)	45	72	29	44	45	
**Education**						
Low (%)	42	11	63	77	46	26.311 (0.000)
Medium (%)	24	33	27	18	25	
High (%)	33	56	10	5	29	
**Household composition**						
Single (%)	32	6	50	55	37	14.595 (0.002)
Couple (%)	68	94	50	46	63	
**Tenure**						
Owner occupied (%)	65	89	59	62	65	5.202 (0.158)
Rented (%)	36	11	41	38	35	
**Urban density**						
High (%)	31	28	12	46	29	21.382 (0.011)
Moderate (%)	29	28	50	18	31	
Low (%)	17	39	21	32	21	
Very low (%)	23	6	18	5	19	
**Volunteers**						
Yes (%)	69	28	58	18	58	27.775 (0.000)
No (%)	31	72	43	82	42	
**Active club member**						
Yes (%)	81	94	97	82	85	6.799 (0.079)
No (%)	19	6	3	18	15	
**Face-to-face contact frequency**						
Number in 2 days	5.51	6.00	4.56	0.00	4.83	25.153 (0.000)
**Perceived loneliness**						
1–5	1.86	1.61	1.93	3.19	1.95	11.319 (0.000)
**Perceived health**						
1–5	3.50	3.94	3.85	3.19	3.57	3.514 (0.016)
**Physically challenged**						
Yes (%)	36	11	18	39	10	8.409 (0.038)
No (%)	64	89	82	61	69	
**Walks**						
Yes (%)	94	100	97	73	93	16.343 (0.001)
No (%)	6	0	3	27	7	
**Uses car**						
Yes (%)	81	100	74	50	78	16.479 (0.001)
No (%)	19	0	27	50	22	
**Uses public transport**						
Yes (%)	45	56	53	9	43	13.016 (0.005)
No (%)	55	44	47	91	57	
**Uses bicycle**						
Yes (%)	80	94	77	50	78	13.334 (0.004)
No (%)	20	6	24	50	22	

## 5. Conclusions

Recognizing the importance of social activities for preventing loneliness and stimulating mental and physical health of senior citizens, this study has examined the heterogeneity in their choices for social activity location types. For this study diary data were collected in 2014 in Noord-Limburg in the Netherlands among 213 respondents aged 65 or over. Based on these data a latent class multinomial logit model was estimated. Three segments were identified, showing different parameters for social interaction locations: home socializers, third place socializers and community center socializers. A fourth segment, non-socializers, consisted of the respondents who did not record any interactions in the two diary days.

Using Chi Square and ANOVA tests, significant differences were found between the segments regarding age, education, income, household composition, volunteering, perceived loneliness and health. The third place socializers are younger, wealthier, healthier, higher educated, and more likely to live with a partner, whereas the non-socializers are older, lower educated, more often single, more often lonely and less healthy. The community center socializers are most likely to have a low income, live alone and live in suburban areas. Regarding mobility the results show significant differences between the segments as well. The results suggest that all third place socializers walk and drive, and the majority cycles and uses public transport, whereas only half of the non-socializers uses a car or bicycle, 9% uses public transport and 73% walks.

There are some shortcomings to this study, some of which might be addressed in future research. Our data collection covered data on face-to-face social contacts at different types of locations. Social interactions other than face-to-face, for instance telephone calls or emails, were not included in this research, whereas these ICT-mediated contacts might play a role in social network maintenance and the prevention of loneliness of older adults. We did not collect detailed information on the social activity locations, such as address or quality of the location, although these location characteristics are likely to affect their use. We did not collect data on the content of the social interaction either. Moreover, the data were collected during the winter, which is likely to have implications for the recorded social behavior. It would be preferable to spread the data collection over all seasons. A final shortcoming of our study is the fact that the sample is relatively small. As a result, some of the segments contain only few observations.

Despite these shortcomings, the findings of this study are relevant for health and social welfare services for older people, local governments and planners. In order to support healthy ageing in place, residential environments should provide the necessary opportunities for social interaction of various segments of the aging population. As indicated by the results, there is a specific segment of lower income elderly for whom the community center seems to be an important location for social contact. While many community centers are disappearing, local policy makers should strive to maintain them to promote social participation of the elderly.

While our latent class analysis focused on the social interactions and their locations, 10% of the sample did not record any social interactions during the two survey days. It is important to pay attention to this segment of elderly, as they are the most vulnerable group, with lower levels of mobility and health and a higher propensity to feeling lonely.

The results suggest that, in addition to personal characteristics, mobility plays an important role in social activity patterns of older adults. The more mobile elderly showed a larger variety of social activity locations. Stimulating their mobility, for instance by providing safe and accessible public transport and walkable neighborhoods, could promote social participation and health of older adults.
